# A Low-Cost, Formaldehyde-Free and High Flame Retardancy Wood Adhesive from Inorganic Adhesives: Properties and Performance

**DOI:** 10.3390/polym9100513

**Published:** 2017-10-16

**Authors:** Shicun Jin, Kuang Li, Jianzhang Li, Hui Chen

**Affiliations:** 1 Key Laboratory of Wood Materials Science and Utilization (Beijing Forestry University), Ministry of Education, Beijing 100083, China; jinsc1994@bjfu.edu.cn (S.J.); kuangli@bjfu.edu.cn (K.L.); 2 Beijing Key Laboratory of Wood Science and Engineering, Beijing Forestry University, Beijing 100083, China; 3 College of Materials Science and Technology, Beijing Forestry University, Beijing 100083, China

**Keywords:** plywood, inorganic adhesive, bonding strength, thermal stability, flame retardancy

## Abstract

Wood composites used in indoor living environments often pose formaldehyde emission and fire hazard problems. In this study, magnesium oxychloride cement-based (MOC) inorganic adhesives are presented as an effective and sustainable binder for plywood applications. The phase composition, microstructure, and thermal stability of the adhesives prepared with different ratios of MgO/MgCl_2_ were investigated. In addition, the dry and wet shear strength and the combustion behavior of the plywood were also examined. The results indicated that the limiting oxygen index (LOI) values of the plywood bonded by the MOC adhesives were higher than those of the plywood bonded by urea-formaldehyde resin. The active MgO/MgCl_2_ molar ratio of 7 was the optimal ratio for the dry and wet shear strength of the plywood with values of 1.02 and 0.88 MPa, respectively, which meet the interior use panel (Type II plywood) requirements. These improvements were ascribed to the increasing ratio of MgO/MgCl_2_ that facilitated the formation of an excellent microstructure. Meanwhile, the continuous hydration phase strengthened the interaction between the MOC adhesive and the wood. With these improved properties, MOC adhesive is expected to be widely used for industrial applications in plywood fabrication.

## 1. Introduction

In recent years, wood composite materials have drawn great interest due to their renewability [1]. In particular, plywood with good mechanical properties has been widely applied in furniture [2,3], residential construction [4] and interior decoration industries [5,6]. Plywood production is mainly dependent on formaldehyde-based resin adhesives at present, which leads to the issue of formaldehyde emission from the panels [7,8]. Moreover, the hot press process has been commonly used for plywood production, resulting in high energy consumption and reduced timber utilization rates [9,10,11].

Magnesium oxychloride cement (MOC) is an air-dried, cement-based, inorganic material prepared with light-burnt magnesia (MgO) powder and magnesium chloride (MgCl_2_) solution [12]. Due to its excellent functional properties, such as fast setting times, extreme fire repellency, low thermal conductivity and high binding potential [13,14], MOC has recently aroused considerable attention and has been widely applied in the rapid repair of infrastructure, such as airport runways and highways [9,15]. The low alkalinity of MOC makes it suitable for mixing with glass fibers to prepare glass fiber-reinforced MOC-based composites to be used in ventilation systems [16], cooling towers [17] and purification tanks [18]. In addition, wood-MOC composites have been found to exhibit superior performance in the already established fields of industrial flooring, grinding wheels, and multifunctional panels, such as decorative, flame retardant, and sound and thermal insulating panels [19,20,21].

In the past decades, there have been many studies which proved that the four primary composition phases of MOC are 2Mg(OH)_2_·MgCl_2_·4H_2_O (phase 2), 3Mg(OH)_2_·MgCl_2_·8H_2_O (phase 3), 5Mg(OH)_2_·MgCl_2_·8H_2_O (phase 5) and 9Mg(OH)_2_·MgCl_2_·5H_2_O (phase 9) in this ternary system of MgO-MgCl_2_-H_2_O [22]. It is worth noting that phases 3 and 5 can form and stably exist at ambient temperatures, while phases 2 and 9 are instable unless temperatures are above 100 °C [23]. It is also accepted that phase 5 crystals can provide the best mechanical properties [24]. Theoretically speaking, the phase 5 crystals can be obtainable from a MgO/MgCl_2_ molar ratio of 5, plus the required water. Because of unreacted MgO particles and the usability of the mixture, excess MgO or H_2_O are used in the production of MOC [15,25].

The application of MOC adhesives in plywood would solve the problems related to formaldehyde emissions, fire hazard, and the energy consumed in plywood production. However, when the ratio of MgO/MgCl_2_ is relatively low, the adhesives might partially penetrate into the wood during the pressing process and result in a less complete hydration phase formation of the plywood interface, which would lead to poor interfacial adhesion strength in the plywood. In addition, it is difficult to spread the MOC adhesives on wood surfaces, which poses a challenge to their application to the plywood. Thus, MOC inorganic adhesives are mainly used in particleboard production and the effect of MOC adhesives on the properties of plywood has been only rarely reported on. It is noteworthy that the ratio of MgO/MgCl_2_ is one of the most critical factors for the hydration product of MOC [26]. The theoretical proportion of MgO/MgCl_2_ needed to ensure the formation of sufficient phase 5 crystals is difficult to determine, since crystal formation can be affected by the reactivity of MgO. However, it is essential to know the optimal molar ratio of MgO/MgCl_2_ before creating further formulations with various additives and fillers for use in commercial products. So far, no clear guidelines are given regarding the optimal molar ratio of MgO/MgCl_2_ needed to achieve the best bonding performance of MOC cement. Therefore, it is essential to examine a very wide range of ratios of MgO and MgCl_2_ and to find the proper molar ratio of MgO/MgCl_2_ in MOC adhesive in order to achieve the best bonding strength in plywood.

In this study, MOC inorganic adhesives with various molar ratios of MgO/MgCl_2_ were prepared and the impact of their microscopic phase behavior on the properties of plywood were observed. These observations are discussed in this paper. The structures and properties of the MOC adhesive, including the crystal phase composition, thermal stability and fracture surface morphology are characterized in detail. The shear strength and flame retardancy of the plywood were investigated by a universal tensile testing machine and a limiting oxygen index instrument.

## 2. Materials and Methods

### 2.1. Materials

The light-burnt magnesia powder, containing 65% reactive MgO by weight, was supplied by Guangzhou Danlin Trade Co., Ltd. (Guangzhou, China). The chemical composition of the solid magnesia powder is shown in Table 1. The magnesium chloride hexahydrate (MgCl_2_·6H_2_O), 98% pure by weight, deionized water and NH_4_Cl (analytical grade) were supplied by Lanyi Chemical Co., Ltd. (Beijing, China). The waterborne polyacrylamide was purchased from Renqiu City Huida Chemical Co., Ltd. (Cangzhou, China). The urea-formaldehyde resin (solid content: 48% and viscosity: 120 mPa·s) was supplied by Jining Hongming Chemical Reagent Co., Ltd. (Jining, China). Poplar veneers (200 mm × 200 mm × 1.5 mm, 8.0% moisture content) were provided by a local plywood plant.

### 2.2. Preparation of MOC Adhesives and Plywood Samples

A specific amount of MgCl_2_·6H_2_O and deionized water were added into a 250 mL beaker and agitated with a magnetic stirrer (RW20 digital, IKA, Staufen, Germany) for 15 min to form a MgCl_2_ solution. Then, 0.05% (by solution weight) waterborne polyacrylamide was added as a thickener and the new solution was stirred at 1000 rpm for 20 min. Thereafter, the light-burnt MgO was added in different amounts and was dissolved at high speed for 2 min to complete the preparation of the MOC adhesives. The molar ratios of active MgO/MgCl_2_ were 3:1, 4:1, 5:1, 6:1, 7:1, 8:1, 9:1 and 10:1. The molar ratio of H_2_O/MgCl_2_ was 18 (including the bound water in MgCl_2_·6H_2_O). For convenience, MOC adhesives with different molar ratios of MgO/MgCl_2_ were named MOC-3, MOC-4, MOC-5, MOC-6, MOC-7, MOC-8, MOC-9 and MOC-10, respectively. The whole reaction process was carried out at room-temperature.

**Scheme 1:**
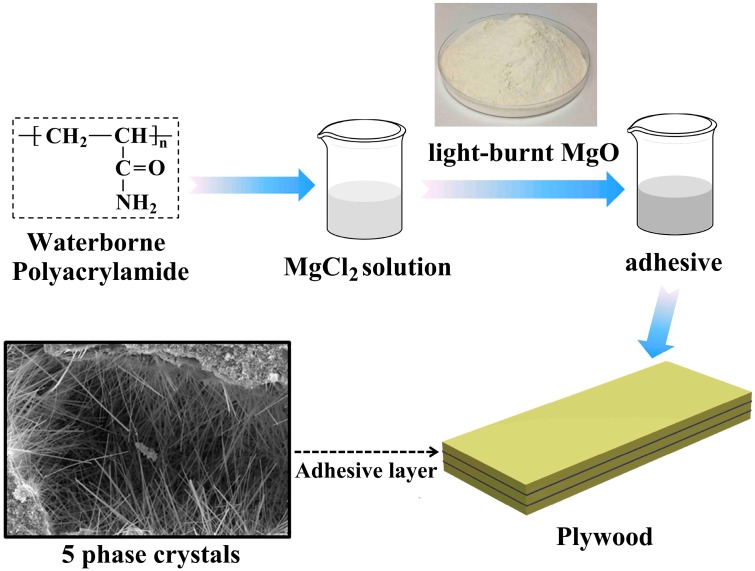
Schematic illustration of plywood bonded with inorganic adhesives.

Three-ply plywood samples were fabricated under the following conditions. The adhesive was applied at a concentration of 250 g/m^2^, which was evenly spread on both sides of the plywood surface using a brush. The coated veneers were stacked manually with the grain direction of each pair of adjacent veneers perpendicular to each other. The plywood samples were cold-pressed using a press machine (CGYJ-100, Shijiazhuang, China) at 3.0 MPa at room-temperature for 24 h. After pressing, the plywood samples were stored at room-temperature for three days before testing. The average thickness of the fabricated samples was 4.552 mm, with a standard deviation of 0.238. A schematic illustration of the plywood bonded by inorganic adhesives is presented in Scheme 1.

### 2.3. Characterization

#### 2.3.1. Viscoelastic Measurement

The viscosity of the freshly prepared MOC adhesives was measured using a rheometer (HAAKE RS1, Berlin, Germany) with a plate fixture (20 mm diameter). The distance was 1 mm for all measurements. The experiments were conducted with a spinning rate of 2 rpm at 23 °C. The data are reported as an average.

#### 2.3.2. Fourier Transform Infrared (FTIR) Spectroscopy

The MOC adhesives were prepared and cast in a cubic silicone mold with dimensions of 40 × 40 × 40 mm^3^. After curing for 24 h at room-temperature, the specimens were removed from the molds and air-cured for 3 days. The cured adhesives were ground into fine powder with a ceramic mortar, then a sample was mixed with folium and KBr at a mass ratio of 1/100 and the mixture was pressed in a special mold. FTIR spectra of the cured MOC adhesives were measured with 32 scans with a Nicolet 6700 spectrometer (Nicolet Instrument Corporation, Madison, WI, USA) set to a wavenumber range from 4000 to 500 cm^−1^ with a 4 cm^−1^ resolution.

#### 2.3.3. X-ray Diffraction (XRD)

After curing for 3 days, the diameters of the particles ground by a ceramic mortar were less than 75 μm. The composition of the adhesives was measured by an X-ray diffraction (XRD) diffractometer (D8 Advance, Bruker, Karlsruhe, Germany), with a cobalt source and a 0.2 theta scan ranging from 5° to 70° at a voltage of 40 kV and a current of 40 mA.

#### 2.3.4. Scanning Electron Microscopy (SEM)

The microstructure on the fractured surface of the cured MOC adhesives was observed by a Hitachi S-3400N scanning electron microscope (Hitachi Science System, Ibaraki, Japan). The surface was sputter coated with 10 nm Au/Pd film prior to examining it under the microscope.

#### 2.3.5. Thermogravimetric Analysis

The thermal properties of the cured MOC adhesives were measured using a thermogravimetric analyzer (TA Q50, TA Instrument, New Castle, DE, USA). About 10 mg of MOC adhesive specimens with 200 mesh (0.075 mm) were weighed in a platinum cup. Thermogravimetry tests were conducted at temperatures of 30 to 800 °C, at a heating rate of 10 °C/min in a nitrogen atmosphere.

#### 2.3.6. Shear Strength

**Scheme 2:**
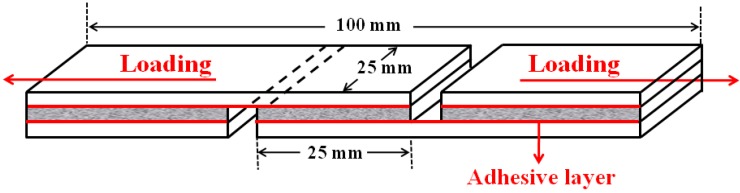
Schematic diagram of the specimen used for the shear strength measurement.

The shear strength demand on the interior use plywood (Type II plywood) was determined according to the procedure described in the Chinese National Standards GB/T 17657 (2013). The specimen (100 mm × 25 mm) was cut from the plywood using a precision saw (ST4, SCM Group, Rome, Italy) at a spindle speed of 5000 rpm. The cut specimens were used to measure the dry shear strength, or were immersed in water at 63 ± 3 °C for 3 h, then dried at room-temperature for 10 min and tested with the wet shear strength test. The shear strength of the cut specimen was determined using a WDW-50 universal tensile testing machine (Jinan Kai Rui testing machine Manufacturing Company, Jinan, China) operating at a loading speed of 20.0 mm/min. The test was carried out with six samples from each group and the data were averaged within the group. Scheme 2 shows the schematic diagram of the specimen used for the shear strength measurement.

#### 2.3.7. Limiting Oxygen Index

The limiting oxygen index (LOI) values of the all plywood panels were determined using an HC-2 oxygen index meter (Jiangning Analysis Instrument Company, Nanjing, China) in accordance with the standard ISO 4589 method. Fifteen samples with dimensions of 135 mm × 6 mm × 3 mm were tested from each group.

## 3. Results and Discussion

### 3.1. Viscoelastic Measurement

The viscosity of the adhesives determined their penetration and wettability on the surface of the substrate, which had a substantial effect on adhesion [27]. The viscosity of the MOC adhesives is shown in Table 2. The MOC-3 adhesive had the lowest viscosity, which was because it had less MgO and generated weaker intermolecular forces in the MgO-MgCl_2_-H_2_O system. As the molar ratio of MgO/MgCl_2_ increased from 3 to 10, the initial viscosity of the adhesive increased from 1653 to 15749 mPa·s. This increase was due to the increasing friction between the molecules in the MOC adhesive [28]. In general, the viscosity required for most wood laminating purposes (both cold and hot press) with wood composites ranges from 5000 to 25,000 mPa·s [29]. As seen in Table 2, the viscosity of MOC-3 to MOC-5 fell below the lower limit, while the viscosity of MOC-6 to MOC-10 showed an acceptable viscosity and was suitable to be used as wood adhesive.

### 3.2. FTIR Spectroscopic Analysis

**Figure 1:**
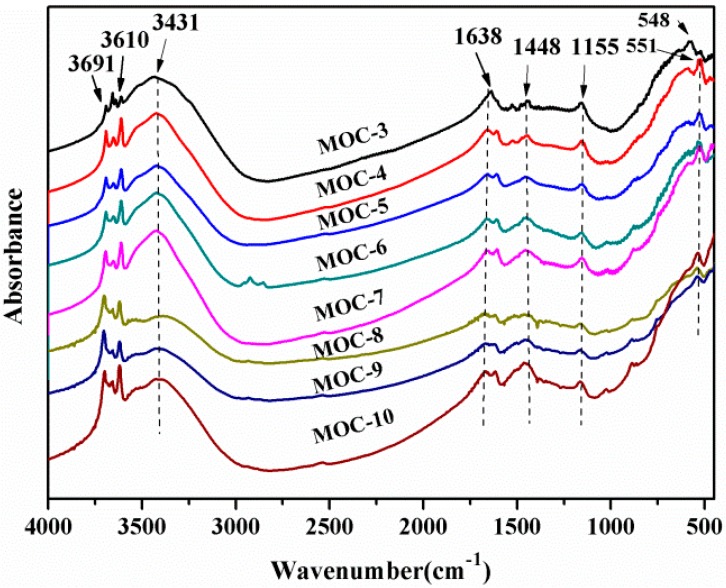
Fourier Transform Infrared (FTIR) spectra of MOC adhesives.

To investigate the component changes in MOC adhesives with each increase of the molar ratio of MgO/MgCl_2_, the FTIR spectra of different adhesives were recorded in the 500–4000 cm^−1^ region and presented in Figure 1. A small peak observed at 3691 cm^−1^ was attributed to the bending vibration of Mg–OH on their structure groups [30]. The bands at 3400 to 3600 cm^−1^ were attributed to the O–H bending vibrations of crystallization water [31]. The peak in the region 1600–1670 cm^−1^ belonged to the H–O–H bending motion [24], indicating the existence of more crystalized water when the samples were prepared at higher ratios of liquid to powder. The bands at 1448 cm^−1^ were attributed to the stretching vibration of C–N [32]. In addition, the 1155 cm^−1^ absorption bands were due to the weak bonding vibration of Cl–O, and the peak at 500–600 cm^−1^ was attributed to the lattice stretching vibrations of Mg–OH [25]. In the case of n(MgO)/n(MgCl_2_) > 7, the intensities of the O–H bending vibrations at 3400 to 3600 cm^−1^ gradually dropped, which was due to the reduction of hydration products [33]. Because of the further addition of light-burned MgO into the adhesive system, two absorption bands in the region 1600–1670 cm^−1^ appeared in the spectra of the sample from MOC-4 to MOC-10, but only a single absorption band appeared in the spectrum of MOC-3. In the case of n(MgO)/n(MgCl_2_) > 5,the intensities of the C–N stretching vibrations at 1448 cm^−1^ gradually increased. This feature was due to the increased waterborne polyacrylamide in the system [34]. It is worth noting that the Mg–OH stretching vibration shifted from 548 cm^−1^ (MOC-3) to 551 cm^−1^ (MOC-4 to -10), likely indicating that these samples had denser structure than MOC-3. This dense structure was caused by a sufficient hydration reaction in this ternary system of MgO-MgCl_2_-H_2_O [35].

### 3.3. X-ray Diffraction

**Figure 2:**
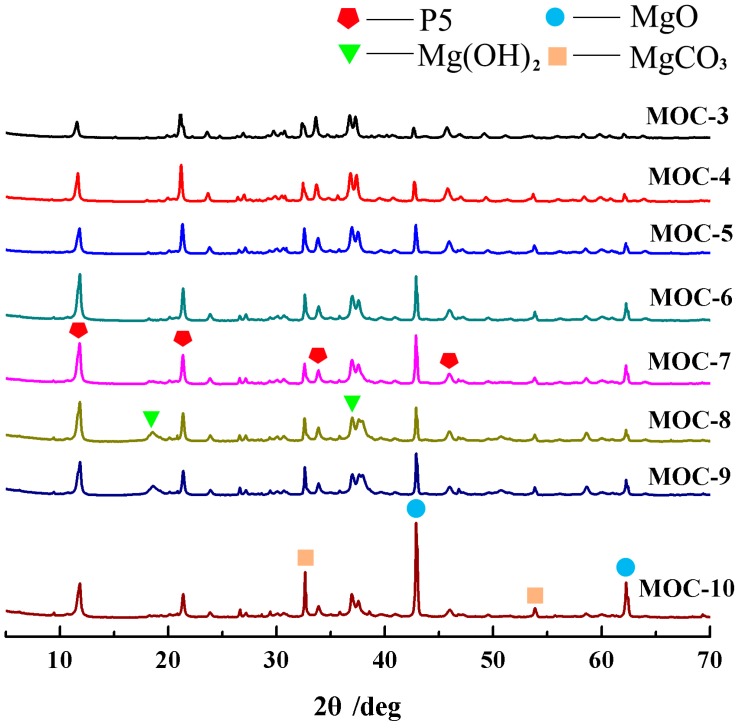
XRD patterns of MOC adhesives.

The effects of the molar ratio of MgO/MgCl_2_ on the cured MOC structure conformations were analyzed by XRD tests (Figure 2). For all MOC adhesives, mineralogical phases were made up of major phase 5, minor MgCO_3_, Mg(OH)_2_ and MgO. Among these components, phase 5 and Mg(OH)_2_ resulted from the formation of hydration products, while other phases originated from light-burnt MgO [36]. As seen in Figure 2, the intensity of MOC-7 at 2θ = 11.8° was higher than that of MOC-3. However, the intensity of MOC-8 became weak, indicating that the amount of phase 5 increased firstly and then decreased. It was observed that phase 5 was the most favorable resultant. With the increase of MgO content, XRD patterns of the MOC-8 and MOC-9 showed a new peak of Mg(OH)_2_ at 2θ = 18.3°, which was caused by the fact that the main reaction of the MgO-MgCl_2_-H_2_O system was altered from a neutralization reaction to hydration reaction with the existence of excessive MgO and water [37]. In addition, the intensity of 2θ peaks at about 42.5° and 61.8° gradually increased after the incorporation of MgO, which was due to the existence of unreacted MgO. According to previous studies [23], the hydration products derived from the neutralization reaction were prone to dissolve into the liquid, bringing about new surface availability on MgO particles, which led to an ongoing neutralization reaction. However, the hydration reaction was able to generate insoluble Mg(OH)_2_ that covered the surface of MgO particles, which impeded the next step of the reactions. From these analysis results, one could come to the conclusion that the neutralization reaction was favorable to the hydration reaction.

### 3.4. Microstructure

**Figure 3:**
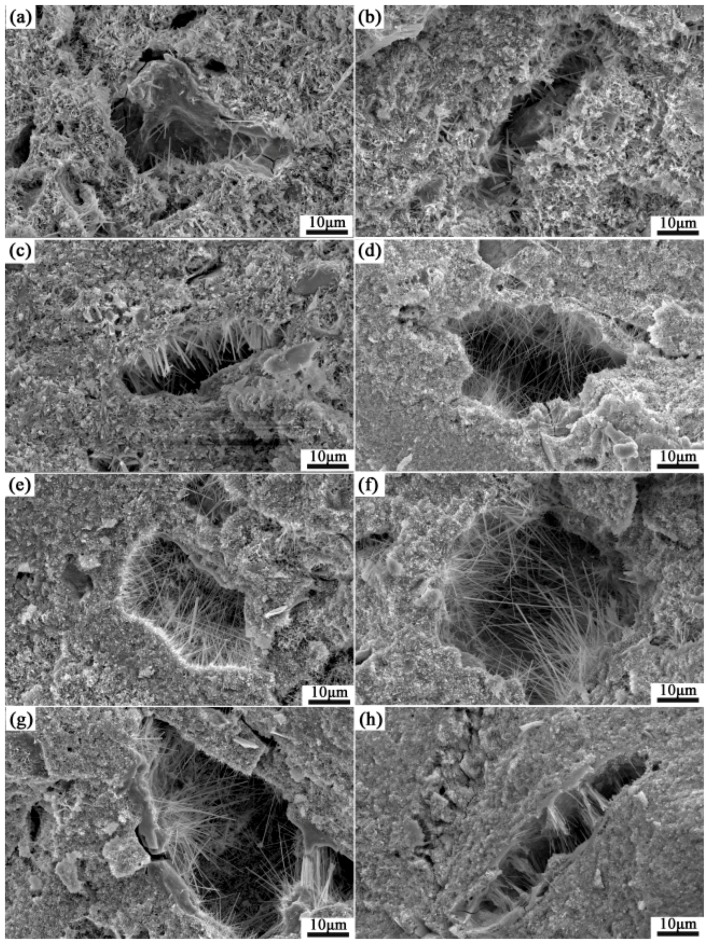
SEM images of the fracture surface of (**a**) MOC-3 adhesive; (**b**) MOC-4 adhesive; (**c**) MOC-5 adhesive; (**d**) MOC-6 adhesive; (**e**) MOC-7 adhesive; (**f**) MOC-8 adhesive; (**g**) MOC-9 adhesive; (**h**) MOC-10 adhesive.

The fracture surface morphologies of the cured MOC adhesives were observed by SEM micrographs. Irregular holes were observed on the fracture surface of the cured adhesives (Figure 3a,b). The images showed loose and disordered structures, because of excess water evaporation in the inorganic adhesive system. As the molar ratio of MgO/MgCl_2_ increased, the fracture surface of adhesives became more continuous, indicating that the structures were denser (Figure 3c–e). This result was attributed to the formation of more crystalline phases. Meanwhile, the unreacted MgO in the system was much more porous than the MgO crystal [19]. The reduction of the invisible capillary channel openings, crystal interfaces and open pores were realized by the addition of MgO. MOC-3 and MOC-4 exhibited amorphous and gel-like hydration products, meanwhile, MOC-5 to -10 showed the plate-like crystal of phase 5 in the matrix. The crystallinity of the gel-like phase 5 was lower than that of plate-like crystal, which caused the lower lattice energy of the product [37]. This result led to a decrease in the thermal stability as well as poor bonding strength. The increase of MgO molar concentration showed a simultaneous increase in the amount of needle shaped crystals in the pores. Meanwhile, the sizes of the needle shaped crystals were more slender, facilitating a strong adhesion effect on the plywood. However, the addition of excess MgO reduced needle-like whiskers (Figure 3f–h), which in turn resulted in a decrease in the bonding strength. The presence of the crystals (needle rod-like phase 5 crystals) indicated the existence of the hydration layer [38]. The stacking thickness of the hydration layer might have formed a lamellar structure on the surface and did have a significant impact on the amount of these hydration products [39] which resulted in the differences in the bonding strength.

**Figure 4:**
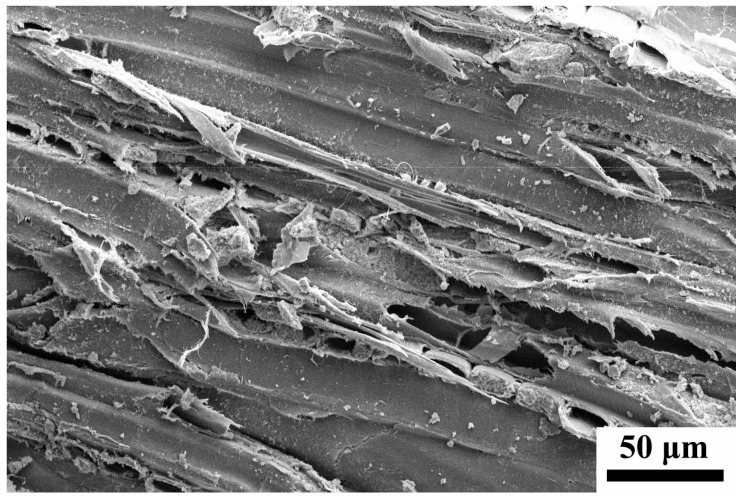
SEM micrograph of the fracture surface of the wood surface bonded by the MOC-7 adhesive.

The bonding performance of the MOC-7 adhesive was analyzed by the SEM micrograph on the fracture surface of the wood surface (Figure 4). It could be observed that the crack extended to the wood cavity and adhesive layer. In general, cohesive failure denoted good bonding performance [4]. The cohesive failure indicated that there was a favorable interaction between the wood cells and the MOC inorganic adhesive, which allowed the wood to be bonded effectively by the adhesive and further increased the wet shear strength of the plywood.

### 3.5. Thermal Stability

**Figure 5:**
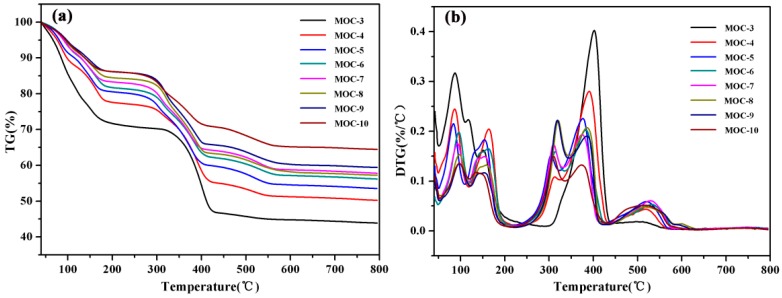
The (**a**) thermogravimetric (TG); and (**b**) derivative thermogravimetric (DTG) curves of the MOC adhesives.

Figure 5 shows thermogravimetric (TG) and derivative thermogravimetric (DTG) curves of the MOC adhesives. The corresponding data are listed in Table 3. The thermal degradation process of adhesives could be divided into three stages. In the first stage, the weight loss prior to 200 °C was primarily related to the evaporation of superfluous water [22]. The second stage, beyond 300 °C, was the loss of the crystallization water of the MOC to give MgO and MgCl_2_ [35]. The final stage, around 500 °C, was mainly related to the decomposition of MgCO_3_ [33]. As can be seen in Figure 5a, with the increase of MgO, the thermal stability of the adhesive was gradually improved. In Table 2, MOC-3 adhesive showed the largest weight loss rate, which was considered to be mostly excess water in the system that was not involved in the hydration reactions and the neutralization reactions [40]. MOC-10 adhesive exhibited the least weight loss rate, which was due to the excessive presence of MgO in the adhesive [41].

As shown by the DTG curves, the temperature at the maximum degradation rate (*T*_max_) of MOC-3 at 86.84 and 401.89 °C and the endothermic peaks intensity were gradually decreased with increasing the addition of MgO, suggesting improved thermal stability of the MOC adhesive. In addition, there were two characteristic peaks in the range of 300–550 °C from MOC-4 to MOC-10—rather than the only endothermic peak of MOC-3—which might be caused by the different microstructures of the reaction products [39]. The degradation peak of MOC-3 at 509.28 °C disappeared, which was due to the fact that there was no residual MgO [42].

### 3.6. Plywood Performance

**Figure 6:**
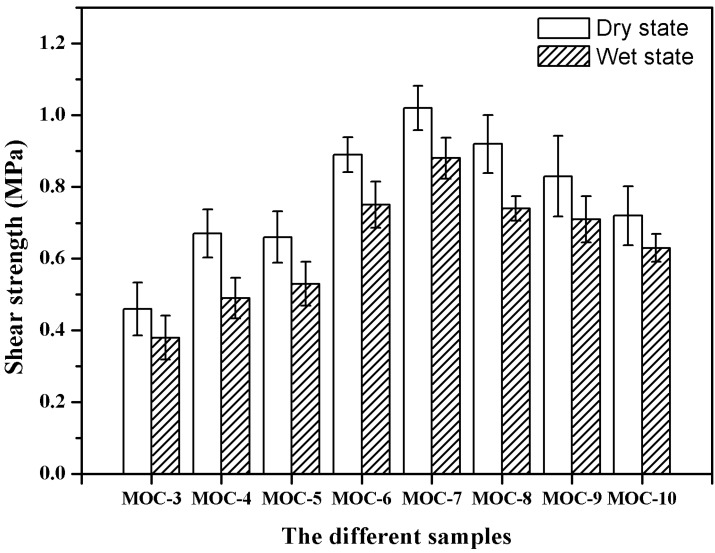
The shear strength of plywood bonded by the MOC adhesives.

As an inorganic binder, cohesion and adhesion were two essential properties of MOC. The cohesion required that the crystalline phase formed continuous structures, and the adhesion relied heavily on the molecular attractive forces between the adhesives and the surfaces of the substrates [43]. Figure 6 shows the adhesion effects of plywood using different adhesives. The shear strength data are shown in Table 4. With the increase of MgO, the shear strength of plywood increased firstly and then decreased, in both the dry and wet states. This correlation generally existed in the adhesives that were based on interparticle interactions. As can be seen in Table 4, the plywood bonded with MOC-3 adhesive exhibited a low dry and wet shear strength of 0.48 MPa and 0.38 MPa, respectively. These measurements were much lower than the interior use panel adhesion strength requirement (≥0.7 MPa). Further addition of MgO was employed to improve the adhesion strength of MOC adhesive in a vertical direction, compared to the substrate. In comparison with MOC-3, the dry and wet shear strength of the plywood bonded with MOC-7 increased by 112.5% and 131.6% to the maximum of 1.02 and 0.88 MPa, respectively, which met the requirements of wood panel bonding strength for interior use. This increase was due to the fact that the addition of MgO facilitated the formation of a dense microstructure [12]. Meanwhile, the continuous hydration phase strengthened the interaction between the inorganic adhesive and the wood. SEM images of the fracture surface of the MOC adhesives showed that there was an integrated and dense structure as the composition increased, which indicated a homogenous and adequate reaction of the adhesive system and served to increase the mechanical performance of the plywood. This result was likely caused by the unreacted MgO and redundant impurities such as SiO_2_, MgCO_3_ and CaCO_3_ in light-burned MgO which played an important role of filler in the MOC adhesive [21]. With further increases to the amount of MgO, the dry and wet shear strength of plywood continuously decreased to 0.72 and 0.63 MPa, respectively. The results were attributed to the possibility that the excessive MgO might lead to an increase in the crystalline stress of the system and further reduced the bonding strength of the plywood [44]. Moreover, for each plywood sample, the wet shear strength of the plywood was slightly decreased compared with the dry shear strength of the plywood, which indicated that the MOC adhesives had a favorable water resistance. Compared to the soy flour adhesive with a wet shear strength of 0.35 MPa [38], the bonding strength of the plywood bonded by MOC inorganic adhesive was obviously higher. Moreover, the maximum wet shear strength of the plywood bonded by MOC-7 (0.88 MPa) was comparable to that of lignin-phenol-formaldehyde resin (0.89 MPa) [39]. In addition, in comparison with the hot press treatment during the fabrication of plywood, the cold press process in this study could save energy and avoid increasing internal stress caused by water vaporization during the hot press process. The wet shear strength of this MOC inorganic adhesive was comparable to that of conventional formaldehyde-based adhesives and other eco-friendly wood adhesives reported in the literature (parameters of different adhesives shown in Table 5) [3,11,29,38,39].

### 3.7. Flame Retardancy of Plywood

The flame retardancy of plywood is critical in the construction industry and in furniture manufacturing in order to avoid possible heavy casualties or expensive property damage caused by fire accidents [41]. However, this characterization was readily ignored in the reported studies. This study used an LOI test to evaluate the flame retardancy of the different plywood samples. The results are shown in Table 6. For comparison, a sheet of plywood bonded by commercially available urea-formaldehyde resin was prepared under the same conditions, and its LOI value was tested. Clearly, the LOI values of the plywood bonded with MOC-3 and MOC-4 were slightly lower than that of urea-formaldehyde resin. When the molar ratio of active MgO/MgCl_2_ was more than 4, the LOI values of the plywood bonded with MOC inorganic adhesives were higher than that of the urea-formaldehyde resin. The MOC-5 adhesive showed an LOI value of 25.7%; as the molar concentration of MgO was increased, the LOI value of the MOC adhesive was likewise increased, especially for the MOC-10 (27.8%). This increase was associated with the growth of hydration products and the unreactive MgO [45]. In short, plywood bonded with MOC inorganic adhesive had a favorable flame resistance threshold. Therefore, it shows great potential for industrial construction applications.

## 4. Conclusions

In conclusion, a low-cost, formaldehyde-free and high flame retardancy wood adhesive prepared from MOC inorganic adhesive was developed via a simple method. The effect of the molar ratio of MgO/MgCl_2_ on the bonding performance of the MOC was examined in this study. The dry and wet shear strength of the plywood increased with MgO content until the MgO content reached the molar ratio of MgO/MgCl_2_ of 7. This increase was attributed to the following reasons: (1) the addition of MgO facilitated the formation of a dense microstructure, (2) a continuous hydration phase strengthened the interaction between the inorganic adhesive and the wood, (3) the appropriate viscosity benefited the even spread of adhesives and formed a stronger interaction between the wood cells and the MOC adhesive. Moreover, our findings show that the plywood bonded with the MOC adhesive with a molar ratio of 7 had the highest dry and wet shear strength, with values of 1.02 and 0.88 MPa, respectively, which meets the requirement for interior use plywood bonding strength. The viscosity of the resultant adhesive was 7126 mPa·s, which suggests that this MOC adhesive is suitable to be used as a wood adhesive. In addition, the MOC adhesive exhibited superior thermal stability (mass loss ratio 41.56% at 700 °C), and the plywood bonded with MOC adhesive showed favorable flame retardancy. With these excellent properties, this MOC adhesive derived from environmentally innocuous resources might offer a valuable innovation to be applied in the plywood manufacturing industry and could replace the formaldehyde-based adhesive.

## Figures and Tables

**Table 1:** Chemical compositions of the solid magnesia powder.

Material	MgO	SiO_2_	Fe_2_O_3_	CaO	Eu_2_O_3_
Magnesia	94.38%	1.54%	2.84%	1.06%	0.18%

**Table 2:** The measured viscosity of the different Magnesium oxychloride cement (MOC) adhesives.

Adhesives	MOC-3	MOC-4	MOC-5	MOC-6	MOC-7	MOC-8	MOC-9	MOC-10
Viscosity (mPa·s)	1653	1821	3463	5971	7126	7930	11,410	15,749

**Table 3:** Thermo-degradation data of the MOC adhesives.

Samples	T_max_ of Thermal Event (°C)					Mass Loss Ratio (%) at 700 °C
	First	Second	Third	Fourth	Fifth	
MOC-3	86.84	151.58	-	401.89	509.28	55.64
MOC-4	83.52	160.65	309.63	387.52	517.59	49.07
MOC-5	84.27	154.60	305.85	376.93	522.13	45.82
MOC-6	85.79	152.33	303.58	373.15	523.64	43.18
MOC-7	86.54	151.06	304.34	370.13	525.16	41.56
MOC-8	85.03	147.04	308.87	373.91	509.28	42.32
MOC-9	89.57	145.53	311.14	377.69	510.79	40.02
MOC-10	94.10	144.77	307.36	371.64	515.33	35.13

**Table 4:** Dry and wet shear strength data of the plywood samples.

Adhesives	MOC-3	MOC-4	MOC-5	MOC-6	MOC-7	MOC-8	MOC-9	MOC-10
Dry strength (MPa)	0.48	0.67	0.66	0.89	1.02	0.91	0.83	0.72
Wet strength (MPa)	0.38	0.49	0.53	0.75	0.88	0.74	0.71	0.63

**Table 5:** Wet shear strength of the wood adhesives reported in the literature [3,11,29,38,39].

Structure	Wet Shear Strength (MPa)	Reference
Soy flour adhesive	0.35	[38]
Condensed tannins-SPI adhesive	0.86	[3]
blocked isocyanates-Starch adhesives	0.73	[29]
Lignin-phenol-formaldehyde adhesives	0.89	[39]
Polysaccharide-SPI adhesive	0.85	[11]
MOC inorganic adhesive	0.88	this work

**Table 6:** Limited oxygen index (LOI) data of the plywood samples.

Samples	Treatment	LOI (%)
A	Urea-formaldehyde resin	24.8 ± 0.02
MOC-3	n(MgO):n(MgCl_2_) = 3:1	23.9 ± 0.02
MOC-4	n(MgO):n(MgCl_2_) = 4:1	24.5 ± 0.01
MOC-5	n(MgO):n(MgCl_2_) = 5:1	25.7 ± 0.01
MOC-6	n(MgO):n(MgCl_2_) = 6:1	26.2 ± 0.02
MOC-7	n(MgO):n(MgCl_2_) = 7:1	27.4 ± 0.01
MOC-8	n(MgO):n(MgCl_2_) = 8:1	27.2 ± 0.02
MOC-9	n(MgO):n(MgCl_2_) = 9:1	27.5 ± 0.02
MOC-10	n(MgO):n(MgCl_2_) = 10:1	27.8 ± 0.01
